# An Endorsement

**Published:** 1914-05

**Authors:** 


### Department of Eugenics.

Conducted by Dr. Malone Duggan and Dr. Theo. Y. Hull.

### THE TEXAS STATE SOCIETY OF SOCIAL HYGIENE.

Headquarters: San Antonio, Texas.
DR. MALONE DUGGAN, President, San Antonio.
DR. F. E. DANIEL, 1st Vice President, Austin.
PROF. A. CASWELL ELLIS, 2d Vice President, Austin.
MRS. ELI HERTZBERG, 3d Vice President, San Antonio.
DR. THEO. Y. HULL, Secretary, San Antonio.
MR. W. L. EVANS, Treasurer, San Antonio.

EXECUTIVE COMMITTEE.

| | | |
|---|---|---|
| Dr. Malone Duggan, Chm. | Hon. J. C. Willacy. | Dr. W. A. King. |
| Dr. Theo. Y. Hull, Sec'y. | Mr. W. L. Evans. | Dr. J. W. Torbett. |
| Dr. W. F. McCaleb. | Dr. Frank D. Boyd. | Dr. F. C. Walsh. |
| Hon. C. H. Jenkins. | Dr. J. R. Nichols. | Dr. Joe Reuss. |
| Rev. A. W. S. Garden. | Dr. J. M. McCutchan. | Dr. Frank Paschal. |

---

### An Endorsement.

The Conference of the American Medical Association on Medical Legislation endorses the following program in the fight against Venereal Diseases and the Sex Question:

(1) Efforts to obtain the co-operation of physicians in reporting venereal diseases, and utilizing their opportunity as advisers in their family practice and advocating publicly a single standard of morals. (2) The encouragement of diagnostic and advisory work like that which has been so successfully done by the New York Health Department and the Oregon State Board of Health. (3) Scientific constructive educational lectures which have been conducted by social hygiene socieites for selected groups of shop-workers, department-store girls and other similar groups. (4) The development of serious consideration of the problem by associations of parents and teachers under the co-ordinated guidance of medical and moral professional auspices. (5) The promotion of suppression and gradual eradication of all the agencies favoring sex immorality and contributory low standards of morals. (6) Constructive efforts to give in normal schools and universities definite information on the sex problems as it will be met by teachers in the course of their school work.

